# What can volumes reveal about human brain evolution? A framework for bridging behavioral, histometric, and volumetric perspectives

**DOI:** 10.3389/fnana.2014.00051

**Published:** 2014-06-25

**Authors:** Alexandra A. de Sousa, Michael J. Proulx

**Affiliations:** Crossmodal Cognition Lab, Department of Psychology, University of BathBath, UK

**Keywords:** cognitive evolution, brain evolution, primates, illusions, histology, brain volume, visual cortex

## Abstract

An overall relationship between brain size and cognitive ability exists across primates. Can more specific information about neural function be gleaned from cortical area volumes? Numerous studies have found significant relationships between brain structures and behaviors. However, few studies have speculated about brain structure-function relationships from the microanatomical to the macroanatomical level. Here we address this problem in comparative neuroanatomy, where the functional relevance of overall brain size and the sizes of cortical regions have been poorly understood, by considering comparative psychology, with measures of visual acuity and the perception of visual illusions. We outline a model where the macroscopic size (volume or surface area) of a cortical region (such as the primary visual cortex, V1) is related to the microstructure of discrete brain regions. The hypothesis developed here is that an absolutely larger V1 can process more information with greater fidelity due to having more neurons to represent a field of space. This is the first time that the necessary comparative neuroanatomical research at the microstructural level has been brought to bear on the issue. The evidence suggests that as the size of V1 increases: the number of neurons increases, the neuron density decreases, and the density of neuronal connections increases. Thus, we describe how information about gross neuromorphology, using V1 as a model for the study of other cortical areas, may permit interpretations of cortical function.

## Background and purpose

Overall brain size has been found to predict cognitive ability in primates (Deaner et al., 2007; MacLean et al., 2014). A similar trend is apparent from the hominin fossil and archeological records: species mean brain size (estimated from fossil endocrania) increases in concert with evidence for increasingly complex behaviors (de Sousa and Cunha, 2012). Can cortical area volumes reveal more about neural function? The cerebrum is the most enlarged part of the human brain. Within it, cortical areas are functional units which can be defined using several additional criteria (topography, connections, histology, and development) with support from geometric coordinates and sulcal landmarks. The physiological relevance of cortical area volumes is of great interest to evolutionary neuroanatomy. Information about the functional relevance of cortical area volumes is necessary for even the broadest interpretations of cortical function on the basis of hominin fossil endocast shape (de Sousa et al., 2010a). Cortical volumes are increasingly available as tools now assist with or fully automate the parcellation of the cerebral cortex on the basis of histology (Schleicher et al., 1999), gyrification (Destrieux et al., 2010), and connectivity (Johansen-Berg et al., 2004).

Although studies proposing a physiological link between brain structure size and function are numerous, only rarely are mechanisms implicated to explain such links. Numerous studies have suggested evolutionary relationships between the absolute or relative sizes of brain structures and various estimates of social, ecological, and sensory factors (For a review see Healy and Rowe, 2007). Sometimes the links are relatively obscure, with neocortex/brain ratio (Dunbar, 1992) and facial motor nucleus volume (Dobson and Sherwood, 2010) linked to social group size, and neocortex/brain ratio and hippocampus volume linked to executive function (Shultz and Dunbar, 2010). In other cases, the sizes of sensory and motor areas are linked to sensory and motor structures, functions, or opportunities; for example, primary visual cortex size is related to an ecosystem rich in light, as diurnal primates have larger primary visual cortices (V1) than nocturnal ones (Barton, 2006), whereas some ophthalmic subterranean mammals lack any cortex with visual function (Bronchti et al., 2002). Our lack of understanding the meaning of cortical area volumes has led to some criticism of their usage in comparative studies (Roth et al., 2010) and yet these correlative studies continue to link behavior to neuroanatomy in what appears to be a meaningful way. Certainly, there is a functional relationship between brain structure and function, but how is this realized, from the microanatomical to the macroanatomical level?

**Figure 1:**
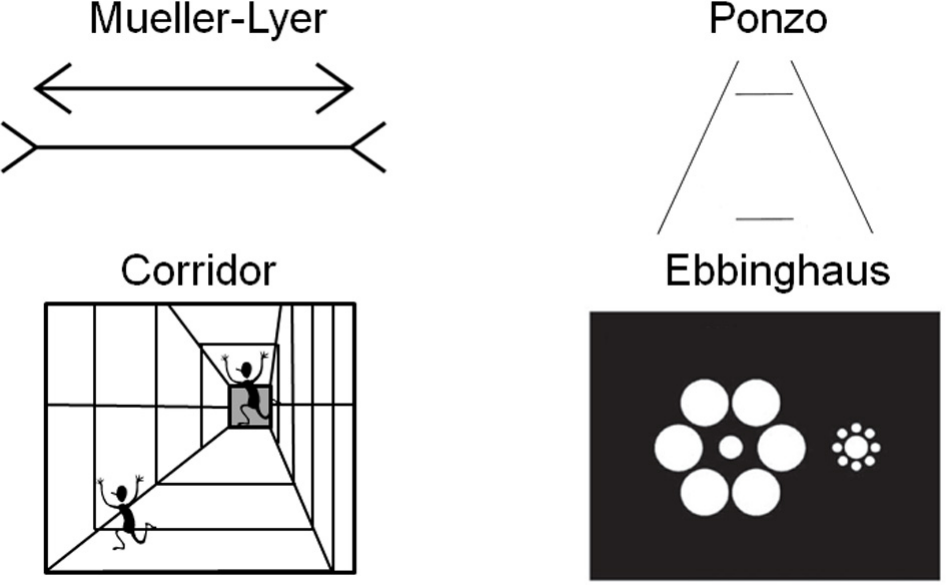
**Examples of four size illusions**. The two horizontal lines are the same size in the Müller-Lyer and Ponzo illusions. The running person is the same size in each part of the corridor illusion. The central discs are the same size in the Ebbinghaus illusion.

A larger cortical area has the potential to process more information than a smaller one. Recent work in human neuroimaging suggests that the size of cortical areas may be relevant for making predictions about perceptual experience (Kanai and Rees, 2011). Individual differences in the surface area of V1 correlate with the perceived strength of two size illusions (the Ebbinghaus illusion and a second illusion they called the Ponzo illusion, although their stimuli were more similar to the Corridor illusion, see Figure 1) (Schwarzkopf et al., 2011). Specifically, the individuals experienced a stronger magnitude of the size illusion when the surface area of V1 was smaller.

Here we extend this idea to research in comparative neuroanatomy where the functional relevance of overall brain size and the sizes of cortical regions have been poorly understood. We outline how the macroscopic size (weight, volume, or surface area) of a cortical region (such as V1) is related to the microstructure of functional brain regions (Kaas, 2000). Next we provide a framework to test this hypothesis by reviewing published data on V1 and visual behavior. Then we make an initial analysis, bringing together for the first time the available neuroanatomical and behavioral data in extant primate species, despite how sparse these data are due to the challenges of such data collection (Tomasello and Call, 2011). To make the connection between structure and function we first build up from the basic physiological constraints at the level of single neurons, and piece together the evidence to reach the level of cortical area volumes and behavior. Then we move down again from the connection between cortical area size and behavior to uncover the microanatomical correlates of visual acuity and the perception of size illusions. The structure-function relationship we propose was first put forward by Elston et al. (1996) and has been developed in part by others (Kaas, 2000; Kanai and Rees, 2011). However, to our knowledge this is the first time that the necessary comparative neuroanatomical research at the macro- and microstructural levels, along with comparative behavioral data, have been brought to bear on the question.

## Linking microanatomy to brain volume

Cortical area size is the label used interchangeably for either surface area, mass, or volume; here we focus on the measure of volume. Volume provides a three-dimensional measure of cortical area size that reflects both the visual field size representation that is primarily mapped by the surface of V1, but also the columnar structure of V1 that includes the feature-specific response properties of receptive fields such as for orientation (Hubel and Wiesel, 1968). Different measures of cortical region size have been shown to have similar structure-function predictive relationships (Kanai and Rees, 2011; Schwarzkopf et al., 2011; de Haas et al., 2012; Lewis et al., 2012). In comparative neuroanatomy, volumetric measurements are common because can they be reliably obtained from histological serial sections more directly than cortical thickness and surface area estimates (Amunts et al., 2007). Volume is a useful measure of cortical region size because the accurate perception of size depends on both the size of the spatial receptive field and the specific representation of visual features such as orientation, and increased volume could afford better processing.

There are biophysical and physiological constraints on the size of functionally-defined cortical areas (Elston et al., 1996). Volumetric or surface area changes in cortical areas indicate an increase in either the size or the number of cells which comprise them. For example, a cortical area might demonstrate a volumetric increase relative to the hypothetical condition of an evolutionary ancestor (Stephan and Andy, 1969; Stephan et al., 1981, 1988). On the one hand, this could be due primarily to a scaling up of neuron size, including a substantial increase in soma volume and an increase in dendrite length. The biophysical properties of the conduction of action potentials make increasing the size of individual neuronal soma less likely. For example, if a neuron's soma were doubled in length its axons and dendrites would also have to double and quadruple in diameter, respectively (Bekkers and Stevens, 1990; Ringo et al., 1994). On the other hand, an increase in the number of neurons may be substantially driving up a structure's volume, in a different way: An increase in neuron number, may be due to the increased biophysical cost of increasing neuronal soma length (Kaas, 2000). This principle has gained insight from recent work demonstrating that for several brain structures, the increase in the mass of that structure across primate species is related to the gaining of neurons. Interestingly, cortical areas gain neurons more quickly than subcortical structures, with V1 gaining neurons faster than the lateral geniculate nucleus and superior colliculus (Collins et al., 2013; Wong et al., 2013).

**Figure 2:**
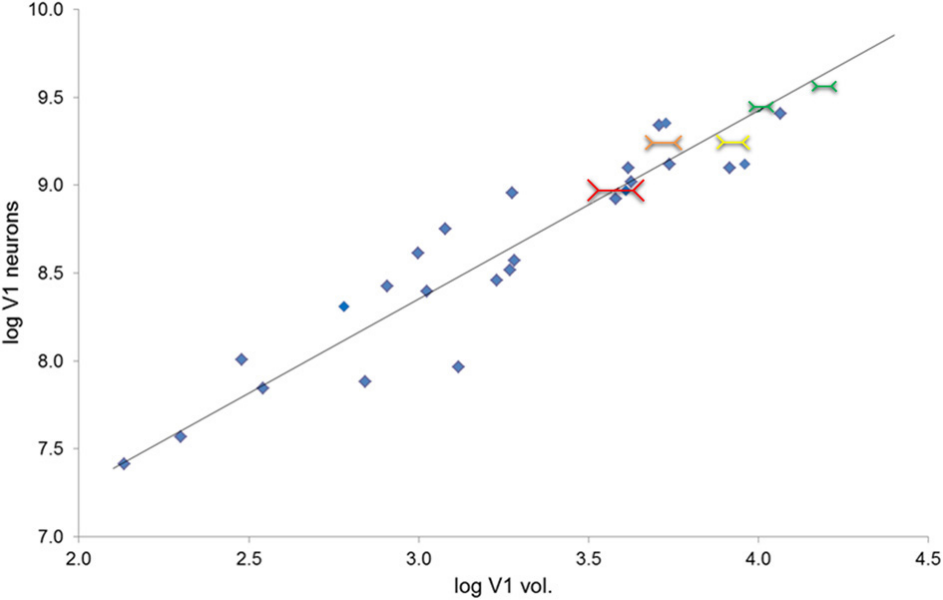
**Total number of V1 neurons increase as a function of V1 volume (*r*_28_ = 0.95); the plot also provides the reduced major axis (RMA) regression of the base 10 logged species mean data (*y* = 1.073x + 5.134, *R*^2^ = 0.899, *p* < 0.001)**. Total neuron number estimated from V1 layer II–VI neuron density times V1 volume in mm^3^ (gray matter only, Frahm et al., 1984; Bush and Allman, 2004; de Sousa et al., 2010a; Lewitus et al., 2012b). (Note that because layer I was not included in the neuron density estimate, these should be considered to be overestimates of total V1 neuron number, but this overestimation is consistent for the whole sample). This figure also depicts visual illusion strength in the context of V1 volume and neuron numbers. Red shows the greatest size illusion experience, followed by orange, yellow, and green as the weakest size illusion experience; the size of the colored Müller-Lyer data points also corresponds to the size of the illusion experience (greater to weakest).

**Figure 3:**
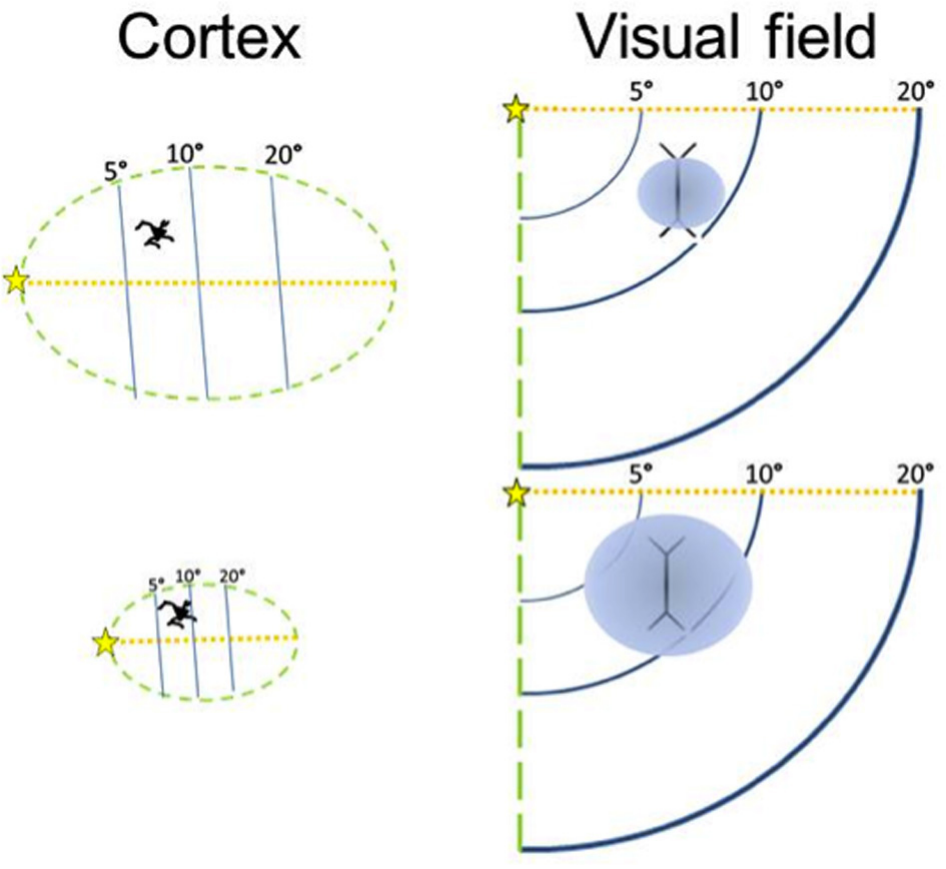
**The size of the visual field represented by a neuron is inversely related to the size of the cortical region**. A larger cortical area has more neurons, although neuronal soma size does not increase much. Modified from Elston et al. (1996).

The volume of V1 in primates ranges from 0.14 cm^3^ in a ~54 g lesser mouse lemur to 15.24 cm^3^ in a ~65,000 g human (de Sousa et al., 2010a). The size of a cortical region is related to body size in so far as brain mass increases with body mass (Jerison, 1955) and cortical region volumes scale to brain volume (Finlay and Darlington, 1995). Also the size of the eye is related to body size, and eye size is also related to V1 size (Stephan et al., 1984; Andrews et al., 1997; Stevens, 2001). It is interesting to note that larger eyes have greater visual resolution than smaller ones (Archer et al., 1999). However, here we suggest why deviations from scaling to such gross variables might occur. The amount of visual cortex required is dependent on the amount of visual input. Our hypothesis focuses instead on how V1 size may be a constraint on image processing. We suggest that, for a given image, the degree of detail which can be represented neurally is dependent on the size of the visual cortex. In primate species, the entire visual field is purportedly mapped retinotopically in V1 (Hubel and Wiesel, 1968) and individual neurons can be examined as having functional specializations related to regions of space, visual features, and other computations (Barlow, 1972). Based on recent work by Collins et al. (2013) larger V1s have more neurons (calculated from their sample, *n* = 6 primates, the least squares slope is 0.8815). Here we approximate this trend for a diverse primate sample (Figure 2; Table 1). Therefore, if visual field size remains constant, the proportion of the visual field that each neuron codes for decreases. Even with changes in visual field size, an increase in neuron number would suggest that each neuron is responsible for a decreased proportion of the visual field. In contrast, given fewer neurons in a smaller V1, each neuron is responsible for coding a greater number of degrees of visual angle of the visual field (see Figure 3).

**Table 1:** **Regressions of number of V1 neurons, number of V1 glia, and maximum visual acuity on V1 (gray matter) volume**.

***y***	***x***	***n***	**R^2^**	***p***	**Slope**	**Low. CI**	**Upp. CI**	**Inter**.	**Low. CI**	**Upp. CI**
**IC OLS REGRESSIONS**										
V1 neurons	V1 vol.	29	0.618	0.000	0.816	0.568	1.064	5.812	5.050	6.575
V1 glia	V1 vol.	29	0.571	0.000	0.999	0.663	1.335	4.919	3.889	5.949
Visual acuity	V1 vol.	11	0.377	0.034	0.459	0.043	0.876	−0.301	−1.538	0.935
**OLS REGRESSIONS**										
V1 neurons	V1 vol.	30	0.899	0.000	1.018	0.886	1.149	5.319	4.873	5.765
V1 glia	V1 vol.	30	0.898	0.000	1.060	0.921	1.198	4.756	4.288	5.224
Visual acuity	V1 vol.	12	0.770	0.000	0.667	0.410	0.924	−0.827	−1.611	−0.043
**RMA REGRESSIONS**										
V1 neurons	V1 vol.	30	0.899	0.000	1.073	0.949	1.213	5.134	4.687	5.580
V1 glia	V1 vol.	30	0.898	0.000	1.118	0.988	1.265	4.560	4.091	5.028
Visual acuity	V1 vol.	12	0.770	0.000	0.761	0.546	1.060	−1.106	−1.891	−0.321

RMA, reduced major axis; IC, independent contrasts; OLS, ordinary least squares; CI = 95% confidence interval; α = 0.05. All data has been log_10_ transformed.

Therefore, the size of a cortical area appears to be inversely related to the proportion of the visual field covered by each of its neurons' dendritic arbors, suggesting that in small areas each neuron takes on a large share of the network (Elston et al., 1996; Elston and Rosa, 1998). It is now well known that a V1 neuron's classic receptive field (the response properties of a neuron, including the region of the visual field for which it codes) is influenced by its lateral interactions with other neurons (Allman et al., 1985a,b).

As V1 increases in volume, dendritic length changes relatively little (Lund et al., 1993; Lyon et al., 1998), so we expect that the proportion of the visual field in which lateral interactions influence the activity of a neuron becomes relatively smaller (Figure 3). This is because the dendrites are not long enough to connect with more distant neurons that represent areas of the visual field that now have a more distant cortical representation in a larger V1. Across the cerebral cortex, the lateral spread of dendrites ranges only by about a factor of two (Lund et al., 1993). Neurons in large areas do not have larger dendritic arbors than neurons in small areas (Tyler et al., 1998), and in fact, the converse is at least sometimes the case, with some smaller cortical areas having neurons with larger dendritic arbors (Elston et al., 1996; Elston and Rosa, 1998). Dendritic length is unlikely to be the main cause of differences in cortical area size across species, either. Crucially, although neurons in the small tree shrew's V1 (surface area = 120 mm^2^) project to over half of its V1 surface, in the larger macaque V1 (surface area = 1200 mm^2^), the neurons project to a relatively smaller region (Rockland and Lund, 1983; Lund et al., 1993).

Note that the size and number of cells other than neurons contribute to cortical area volume. These cells play a supportive role to neurons and their scaling reflects this. We find that glia increase in number with V1 volume (*y* = 1.118x − 4.560, *R*^2^ = 0.898, *r*_28_ = 0.95, *p* < 0.001, two-tailed; Table 1). Similarly, Collins and colleagues reported that cells other than neurons (mostly glia) increase in number in concert with neurons, and in near-linearity with V1 mass (Collins et al., 2013). This is in accordance with the ratio of glia to neurons remaining constant in V1, and not increasing with brain size in primates (Lewitus et al., 2012a).

## Comparative behavioral data

If neurons in a larger V1 provide a more detailed representation of visual space, then changes in the size of V1 should have functional correlates. As a metaphor for these differing representations, consider the number of pixels coding a digital image. A smaller V1 would have fewer pixels to represent an image, and each pixel would sample across a wider context to reach its value. A larger V1 would have more pixels to represent an image, and thus each pixel would provide the value for a small area of space and would not be as influenced by context. V1 volume in particular would also allow for the more accurate coding of feature information in each pixel, such as orientation, again allowing for a more accurate representation of the information in a pixel that would be less influenced by context. This metaphor suggests two potential measures of function: size perception and visual acuity.

The influence of context on perception can be assessed through the use of geometrical illusions. There is a class of visual illusions in which context (i.e., the surrounding background features) affects the perception of the size of an object. Classic size illusions include the Ponzo and Müller-Lyer illusions (see Figure 1). These illusions use the addition of “irrelevant” surrounding spatial information, such as flanking lines, to influence the size perception of the central, “relevant” lines that are tested. The structure of the brain, specifically in this case the size of V1, should have functional implications for processing such stimuli. Indeed this has been demonstrated in humans using measurements of surface area and volume acquired by magnetic resonance imaging (Schwarzkopf et al., 2011). Area V1 is a likely correlate of size perception because the receptive field properties of its neurons code for oriented lines in discrete spatial locations with a retinotopic organization (Hubel and Wiesel, 1968; Schwartz et al., 2002). Higher order areas instead code for more complex combinations of features in larger receptive fields (Gottlieb et al., 1998). Moreover, because the perception of the illusion occurs across the visual field at small spatial scales, other higher order areas (such as inferotemporal cortex) would be unlikely, as would areas that precede V1 in sensory processing such as pre-chiasmatic cells due to their simpler processing and uni-laterality in terms of only processing part of the visual field. This hierarchical organization—in which simple features are processed in primary sensory areas and more complex combinations of features are processed in higher order areas—has been demonstrated by neurophysiological studies and behavioral studies of perception and perceptual learning (for a review see Proulx et al., 2014).

Here we assessed whether V1 volume and visual perception in primates are consistent with the model we have outlined that relates neural structure and function. Primates provide an optimal assessment of the microanatomical, macroanatomical, and psychological aspects of this model due to the convergence of comparative behavioral data (Segall et al., 1963; Fujita et al., 1991; Fujita, 1997; Nakamura et al., 2006; Barbet and Fagot, 2007; Suganuma et al., 2007; Imura et al., 2008; Parron et al., 2008; Pepperberg et al., 2008; Tudusciuc and Nieder, 2010; Schwarzkopf et al., 2011), gross neuroanatomical data and micro-neuroanatomical data (Frahm et al., 1984; Bush and Allman, 2004; de Sousa et al., 2010a; Lewitus et al., 2012b).

We reviewed studies of visual illusion perception in primates to compile quantitative data for comparison of illusion magnitude and V1 volume. We performed a literature search for articles reporting size illusion data in non-human animals. Table 2 displays those studies and size illusions that were found, and also notes those that were suitable due to the reporting of quantitative data that could be used to calculate illusion strength (some studies only presented a qualitative result and therefore were not suitable for the analysis here). A large human literature exists, and a representative cross-cultural study was chosen to represent humans in addition to the human data present in the articles that compared humans with non-human animals. Some size illusions have been under investigation for over 100 years (Müller-Lyer, 1889), and the methods for their study are well-established and similar across the species shown in Table 2. The quantitative magnitude of the size illusion was taken as the percentage difference in the perception of object size caused by the illusion manipulation. The magnitude of the size illusion was expressed in many articles as a percentage difference in object size which was derived from the points of subjective equality on the psychophysical functions. The point of subjective equality indicates the perceived size of the object (manipulated by a size illusion) in comparison to another object when the viewer reports the two to be the same size, when in fact one is larger than the other. If this information was not reported, then the authors were contacted and those authors provided this data upon request. Volumes of V1 for the matching anthropoid species (or where not available, genus) were taken from published datasets (Frahm et al., 1984; Bush and Allman, 2004; de Sousa et al., 2010a; Lewitus et al., 2012b).

**Table 2:** **Comparative behavioral data on the perception of visual illusions by primate and avian species**.

	**Illusion perceived?**			
**Species**	**Ponzo**	**Corridor**	**Mueller-Lyer**	**Ebbinghaus**
Human	Y (Fujita, 1997)	Y (Imura et al., 2008; Schwarzkopf et al., 2011)	Y (Segall et al., 1963; Tudusciuc and Nieder, 2010)	Y (Parron and Fagot, 2007; Schwarzkopf et al., 2011)
Chimpanzee	Y (Fujita, 1997)	Y (Imura et al., 2008)		
Baboon		Y^*^ (Barbet and Fagot, 2007)		N (Parron and Fagot, 2007)
Macaque	Y (Fujita, 1997; Tudusciuc and Nieder, 2010)			
Capuchin			Y (Suganuma et al., 2007)	
Parrot			Y^*^ (Pepperberg et al., 2008)	
Pigeon	Y^**^ (Fujita et al., 1991)		Y/N^***^ (Nakamura et al., 2006)	

* Illusion strength not quantifiable.

** 23.4% in pigeons vs. 11.5% in humans (Nakamura et al., 2006).

*** Inconsistent reports.

Note that humans are difficult to assess with a single marker of size illusion strength, given that the perception of size illusions appears non-existent once environmental experience and culture are considered (Segall et al., 1963), however can also be modulated by cognitive control mechanisms (Proulx and Green, 2011).

We also investigated the relationship between visual acuity and V1 volume in primates. Data were obtained from the literature for visual acuity (Kirk and Kay, 2004) and primary visual cortex volume (Frahm et al., 1984; Bush and Allman, 2004; de Sousa et al., 2010a; Lewitus et al., 2012b). For this sample, data were available for both diurnal and nocturnal primate species, which were plotted separately.

## Linking brain volume to behavior

We examined the number of V1 neurons as a function of V1 volume in primates (data from: de Sousa et al., 2010a; Lewitus et al., 2012b). As shown in Figure 2, the total number of neurons in V1 increases as a function of V1 volume (*r*_28_ = 0.95; least squares regression of the logged species mean data, *y* = 1.073x + 5.134, *R*^2^ = 0.899, *p* < 0.001). Figure 2 also depicts the relationship between V1 volume and visual illusion strength in the context of the relationship between V1 volume and neuron numbers. V1 size appears to be related to visual perception across primate genera; this is due to a negative correlation between the logarithm of V1 volume and illusion strength (*r*_3_ = −0.71; not shown in the figure). This result parallels a study of human inter-individual differences in illusion strength and V1 surface area (Schwarzkopf et al., 2011). The data are consistent with the proximate model outlined above and suggest that anthropoid genera with larger V1 volumes have a weaker experience of size illusions. Consistent with the within-species data for illusion strength and V1 surface area previously reported for humans (Schwarzkopf et al., 2011), V1 size appears to be related to visual perception even at the level of genera across primates.

Might this trend extend beyond anthropoids to include other taxa, such as birds (Fujita et al., 1991; Nakamura et al., 2006, 2009a,b)? The Wulst of the pigeon (187.43 mm^3^ in volume) contains the primary visual area (plus the much smaller primary somatosensory area) and is certainly much smaller in size than human V1 (Jarvis et al., 2005; Reiner et al., 2005; Iwaniuk et al., 2008). In a comparative test of the Ponzo illusion it was found that the pigeon experienced the illusion more strongly (23.4%) than humans (11.5%), consistent with the trend shown in anthropoids (Fujita et al., 1991), and with individual differences in humans (Kanai and Rees, 2011). Although size illusions have not been assessed in larger bird species, it is worth noting that visual acuity has been assessed for a number of birds both anatomically and behaviorally. Visual acuity increases as a function of body and brain mass in birds (Kiltie, 2000), with large birds of prey such as falcons having particularly high acuity (Fox et al., 1976).

**Figure 4:**
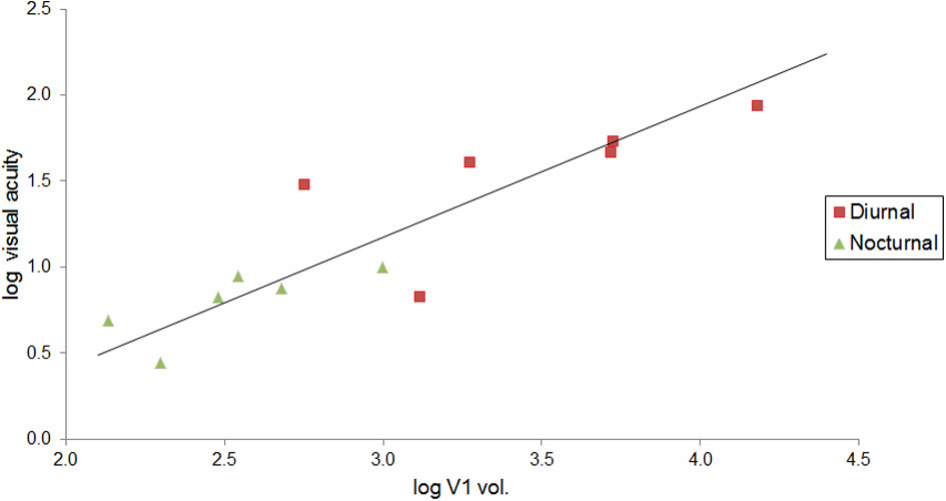
**Visual acuity increases as a function of V1 volume in both diurnal and nocturnal primates**. Log-log (base 10) RMA plot of V1 volume (mm^3^, as above: Frahm et al., 1984; Bush and Allman, 2004; de Sousa et al., 2010a; Lewitus et al., 2012b) as a function of visual acuity (*y* = 0.761x − 1.106, *R*^2^ = 0.770, *p* < 0.001). The species' visual acuity was estimated based on the maximum visual acuity value (c/deg) out of all values listed for behavioral and anatomical visual acuity (Kirk and Kay, 2004).

Visual acuity also increases with V1 volume in primates. Although vision may have an increased effect on brain size scaling with increased chromacy (Barton, 1998), this trend can be seen in Figure 4 with nocturnal and diurnal primates (*y* = 0.761x − 1.106, *R*^2^ = 0.770, *r*_10_ = 0.88, *p* < 0.001, two-tailed). Figure 4 also illustrates how diurnal primates, with the exception of the ring-tailed lemur, all have greater visual acuity than nocturnal primates, and diurnal primates, with the exception of the common marmoset, all have greater V1 volume than nocturnal primates.

## Linking behavior to microanatomy to brain volume

The negative correlation between primary visual area size and size illusion magnitude appears to hold not only for humans, but also across other anthropoids (and potentially birds as well). The positive correlation between primary visual area size and visual acuity converges with these results to suggest the brain volume has implications for function. The microanatomical basis of this relationship can be examined in further detail with additional comparative research.

A key aspect of the model which can be assessed using neuroanatomical data is the perceptual correlate of increasing the number of neurons per degree of the visual angle. Across mammals, larger brains have fewer neurons per unit volume (Tower, 1954). Similarly, across anthropoids, V1 neuron density decreases with brain size (Sherwood et al., 2007). However, V1 volume increases with brain volume at a rate which outpaces the decrease in cortical neuron numerical density. Therefore, we show here that as V1 increases in volume, V1 total neuron number also increases substantially (Figure 2).

When there are functional demands specific to different modules, brain structures may evolve as part of functionally defined modules in a mosaic fashion (Barton and Harvey, 2000), although any changes in brain organization are likely to occur in a similar manner even in distantly related species (Krubitzer and Seelke, 2012). A cortical area under strong selective pressure (such as V1) might exhibit reorganization at both the micro- and macro-anatomical levels. The relationship between microanatomy and macroanatomy would be greatest within a group of brain regions linked by a common modality; such areas would deviate from overall brain scaling trends while remaining similar to each other. In support of this, in a group of higher primates, visual area neuronal volume densities (measured as the Gray Level Index, GLI) scale specifically with the sizes of visual structures, but do not scale with unrelated changes in brain size (de Sousa et al., 2010b). Further, within humans, there is a great deal of intraspecific variability in V1 volume [varying by a factor of up to 3; (Andrews et al., 1997)], something also seen in apes (de Sousa et al., 2010a). However, this variation is specific to the visual system and there is strong correspondence between the sizes of different visual brain structures [V1, LGN, and optic tract; (Andrews et al., 1997)].

Given the negative relationship between brain volume and neuron numerical density, in larger brains neuronal soma are more sparsely distributed, as cortical tissue is increasingly comprised of space for connections (Sherwood and Hof, 2007; Semendeferi et al., 2011; Teffer et al., 2013). Thus a larger V1 has more neurons, yet the lower density also suggests it has less cortical tissue per unit volume occupied by neuronal soma, and more cortical tissue per unit volume available for interneuronal connections (de Sousa et al., 2010b). During development, an increase in neuropil coincides with increased cortical connectivity (Eayrs and Goodhead, 1959; Cragg, 1975). The increased neuropil (and connections) may afford greater neuroplasticity and behavioral plasticity, either by enabling synaptic pruning or alternatively by reflecting new connections (Rauschecker, 1991; Anurova et al., 2014). Plentiful connections might give rise to greater behavioral flexibility more directly, such as the development of a saliency, or attention, map in V1 formed by connections between layer II-III pyramidal cells (Li, 2002).

### Functional utility of illusions

In the case of so-called “illusions” a representation of an object is distorted due to the influence of context. A number of theories propose that visual perception is more accurate when illusion strength is relatively low (Kanai and Rees, 2011; Schwarzkopf et al., 2011). Although this might be true within the confines of a psychophysical task, this perspective might not generalize to perception outside the laboratory where evolutionary pressures led to the neuroanatomical variability present among anthropoids.

At this point we can only speculate about the ecological significance of illusions. The perception of illusions may not be errors, *per se*, and might in fact convey an evolutionary advantage. Possibly, they make for simpler processing of complex stimuli. Faster or simpler perception of stimuli might initiate reflexive motor responses or facilitate decision making (Proulx and Green, 2011) driven by attentional maps in areas such as V1 (Li, 2002). Similarly, in some cases poor visual acuity might be preferred over high visual acuity. Even within the visual field of humans, only the center of focus provides high visual acuity due to the density of cone cells in the fovea of the retina. Peripheral vision is conveyed by rod cells that are less dense. Yet information processing of peripheral information is sufficient for successful navigation and obstacle avoidance in an automatic fashion that does not tax attentional resources.

Further, illusions reveal the influence of the context, rather than purely local information, on perception (Gregory, 1968). The perception of a feature such as size might be influenced by one's goal, such as with the size-weight illusion and the consideration of objects for throwing (Zhu and Bingham, 2011). The most straightforward conclusion relating V1 size to the size perception is that an increase in cortical area size diminishes the role of context on size perception. Even though humans have a larger absolute V1 than other species, they are still susceptible to illusions and even prioritize size on the basis of these illusions, suggesting that the context might be valued for rapid information processing. For example, contextual information provided by illusions initiates a reflexive response which occurs in the absence of awareness (Moore and Egeth, 1997). Also, size generated by the Müller-Lyer illusion can capture attention more strongly than standard size (Proulx and Green, 2011). But, the relatively low illusory strength in humans could indicate that humans process visual stimuli more slowly than many other species.

Cross-culturally in humans, there have been reports that the perception of size illusions appears non-existent once environmental experience and culture are considered, as with extant hunger-gatherer societies (Segall et al., 1963). Although compelling, later work found that the results may have underestimated the perception of size illusions due to a methodological issue; attention to the size illusion stimuli was modulated by cognitive control mechanisms (Gardner, 1961; Davis and Segall, 1971). This suggests a certain degree of perceptual flexibility that big brains might afford. The perception of size and illusions is not always compulsory because cognitive control can modulate the effects of context such that top-down attention can enhance or diminish the influence of context (Gardner, 1961; Davis and Segall, 1971). One way to resolve the problem of whether illusions are errors or not is that perhaps a level of behavioral flexibility is a marker of a larger cortical area, such that context is no longer processed reflexively, but can instead be used or ignored as a function of current goals of an animal such as the primates examined here. This flexibility arises as a function of two further clues to the relation between structure and function in neural basis of size perception: the potential increase in connections between neurons in V1 as it is increased and the flexible role of attention that can take advantage of this scaffolding (Chittka and Niven, 2009).

### Constraint and adaptation in the evolution of brain structures

Our model proposes that there are functional consequences of changes in the absolute sizes of individual topographically-defined brain regions, and thus has implications for the constraints and adaptive pressures involved in the evolution of brain structures. Across mammals, the sizes of individual brain structures are highly linked to total brain volume (Finlay and Darlington, 1995) and there is a relationship between neuron number and brain volume (Haug, 1987). Although brain size can be linked to general aspects of behavior and brain organization, there remain open questions about whether it is relative or absolute brain structure size that is important and how constraints and adaptive processes influence brain organization.

First, there is the issue of relative vs. absolute brain size. V1 volume is the most dramatically reduced cortical area in modern humans (relative to brain size) (de Sousa et al., 2010a). Anthropologists have long debated whether that the relative reduction in V1 volume is linked to overall brain size (Jerison, 1975), or rather due to functionally-relevant expansion of higher order visual and multisensory cortex (Dart, 1925). In fact, human absolute V1 volume is larger than or similar to that of their closest relatives, the chimpanzees and bonobos (de Sousa et al., 2010a). Therefore, for V1, absolute volume might be more informative about visual function than relative volume. Unlike absolute V1 volume, a relative measure of V1 size according to overall brain size does not appear to have the same consistent relationship with illusion strength. These results support the hypothesis that the evolution of particular functional brain regions might be more important for understanding human brain evolution.

Second, the volume of the entire brain is constrained due to its high metabolic costs (Fonseca-Azevedo and Herculano-Houzel, 2012), but energy requirements vary among brain regions (Karbowski, 2007). Therefore, there may be an additional interaction between a given brain region's metabolic cost and the functional relevance of its size. Where there are additional selective pressures to maintain an optimal size, brain structure size might not merely need to keep pace with brain size scaling.

Third, cortical region axis location influences neuron size and number. Our model is based on V1, which is perhaps the largest cortical area in higher primates (Felleman and Van Essen, 1991)—but the brain is not a homogenous tissue and variations exist across cortical zones (Herculano-Houzel et al., 2013). From lower to higher order visual areas, neurons have increasingly large receptive fields; and in crossmodal areas receptive fields bring together multiple sensory maps. Scaling relationships are not identical across the cortex, and moving along a caudal rostral axis in particular cortical areas follow a gradient in neuronal density (Collins et al., 2010) and dendrite complexity (Elston et al., 2009, 2011; Manger et al., 2013).

Fourth, phylogeny is another important influence on neuron number and size. Different mammalian clades show different specific positive scaling relationships between neuron number and brain weight (Herculano-Houzel, 2012; Neves et al., 2014) and neuronal soma volume and brain volume (Haug, 1987; Sherwood et al., 2003; Elston and Manger, 2014).

### Neuroanatomical basis for perception and attention in V1

Computational models of how attention can prioritize the information processing of salient objects in the visual environment build upon the anatomical structure of V1. Our model builds on the work of Elston et al. (1996) who have focused on V1 differences between taxa in layer III pyramidal cells as the specific cells represented in our model in Figure 3. The coding of space has implications for not only perception, as indicated by the acuity and size illusion data reviewed here, but also for attention, the cognitive mechanism of selecting information in primary sensory areas for further processing in higher order areas. On the computational modeling side, work by Zhaoping Li and colleagues (Li, 2002) on the development of a saliency, or attention, map in V1 focuses on the same layer II–III pyramidal cells studied by Elston because these cells are the crucial link to provide contextual modulation of neural activity that represents other portions of the visual field. For example, behavioral evidence has shown that illusory size receives enhanced attentional processing (Proulx and Green, 2011). One hypothesis is that these pyramidal cells in V1 might be a specific link between micro- and macro-structure in comparative neuroanatomy and psychology.

### Implications for the hominin fossil record

The examination of brain structure in primates and understanding its link to perceptual function can provide insight into the evolution of human cognition and consciousness. Brain size has increased throughout human evolution. Although brain size has traditionally been linked to enhanced cognitive function, the hominin fossil and archeological records have documented cognitive advancements, such as tool use, that have preceded major changes in brain size (de Sousa and Cunha, 2012). The suggestion that brain reorganization occurred in early hominins prior to brain size expansion dates to Dart's (1925) description of the Taung juvenile *Australopithecus africanus* endocast—the first australopith ever discovered—and the question of whether brain size and brain organization can evolve independently has had important implications for paleoanthropology. Here the comparative data demonstrate why changes in the sizes of brain structures early in hominin evolution may be functionally relevant.

V1 size does differ within and between extant primate species, however nothing had been known about the implications or causes of V1 size variation between closely related hominin species, for which the link between brain size, brain organization, and brain function remains obscure. This first piece of evidence for constraints and adaptive pressure in the evolution of the hominin brain arose from the location of the lunate sulcus, a marker of the anterior limit of V1 in non-human higher primates. Although the *A. africanus* described by Dart had an ape-like brain size, the lunate sulcus was said to be in a posterior location (a more reliable assessment has been made on a newer discovery; see Holloway et al., 2004). This indicated a smaller V1 and thus a greater proportion of cortex that could be devoted potentially to higher level cognition. Similarly, a recent fossil hominin, *Homo floresiensis*, has an ape-size endocranial volume (426 cm^3^; Kubo et al., 2013) but is associated with modern-like brain organization including a posteriorly positioned lunate sulcus, and surprisingly sophisticated tools (Morwood et al., 2004). Further, large endocranial volumes are known for modern humans (range 1090–1880 cm^3^) and Neanderthals (range 1172–1740 cm^3^) (de Sousa and Cunha, 2012), but it has been suggested that they could have differed in functionally-relevant brain organization. Modern humans, compared to Neanderthals, are estimated to have a slight, statistically insignificant decrease in occipital lobe volume (Balzeau et al., 2012) and based on their smaller orbit size, a decrease in the size of striate and extrastriate areas has been suggested (Pearce et al., 2013). The model here suggests that perhaps changes at the sensory level create the necessary advantages in neural processing that allow for increasingly complex behaviors, followed by a later expansion of higher-level associative areas only possible once the benefits of such behavioral innovations, such as tool use, arise.

The study of extant species provides the tantalizing hypothesis that the reconstruction of the evolution of the brain through endocasts holds the possibility to reveal how fossil hominins saw the world, perhaps despite the pessimism held by some: “It might be interesting to know how cognition (whatever that is) arose and spread and changed, but we cannot know. Tough luck” (Lewontin, 1998).

## Initial clues and remaining questions

The comparative psychological and neuroanatomical data available are consistent with a model that relates primary visual cortex size to visual acuity and the perception of visual illusions. An increase in the size of V1 begets a decrease in the magnitude of the perception of size illusions (Kanai and Rees, 2011). Here we report that overall the neuron volume density decreases with brain size in higher primates. This indicates that as brains get bigger, less space is devoted to neuronal soma and there is more space for connections. This suggests that as the size of V1 increases, the number of neurons increases. Thus a larger absolute V1 can provide the basis for lower illusion strength due to having more neurons to represent a field of space.

Future comparative studies of sensory information processing could be quantified to more easily permit comparisons across species (rather than just testing qualitatively whether the animals can do the task). The mechanism in question might be investigated in further studies which incorporate information about neural circuits (e.g., dendritic arborization, synaptic density), a topic which is revealing itself to be surprisingly complex (Elston et al., 2009).

Does the relationship hold across all species in general, and within primates in particular? Clearly the current literature provides scant data for a full examination of that question. More comparative studies of visual illusions in non-human animals paired with investigations of comparative neuroanatomy can shed light on our understanding of structural and functional relationships and on the evolution of cognition (Emery and Clayton, 2005; de Sousa et al., 2010b; Tomasello and Call, 2011; MacLean et al., 2012).

Importantly there are many novel questions arising from this study that such a research initiative can address. For example, there are many other illusions that are used to assess conscious perception in human and non-human animals and the potential neural basis for these are of great importance as well. A recent study examined illusory size perception in the experience of after images, and found that V1 activity corresponded to perceived size rather than retinal size (Sperandio et al., 2012). Certainly this model need not be restricted to vision, either. The primary auditory and somatosensory cortices can also be assessed through comparative psychological and neuroanatomical studies as well. Given the recent findings that compare visual to auditory neuron numbers and area mass in non-hominid primates (Collins et al., 2013; Wong et al., 2013), it would be interesting to examine neuronal size, neuron density, and area mass, volume, and surface area for such future studies. The approach of the model presented here is most relevant for assessing functional changes in sensory, rather than association areas. For example, the addition of novel association areas seems to be another way that brains expand in size and diversify in function (Kaas, 1989, 2000; Kaas et al., 2013). However, although the details would be harder to examine, even high level multisensory areas, such as those in the superior temporal sulcus and posterior parietal cortex (Pasqualotto and Proulx, 2012; Proulx et al., 2014), are involved in multisensory illusions (McGurk and MacDonald, 1976) and cortical size could reflect this. This bottom-up approach to understanding brain evolution, that is from a sensory and perceptual perspective rather than a top-down or high-level cognitive and linguistic perspective, might yield the sorts of findings that have hitherto been overlooked in the investigation of cognitive evolution and the origins of consciousness (Humphrey, 2011; Proulx, 2011).

### Conflict of interest statement

The authors declare that the research was conducted in the absence of any commercial or financial relationships that could be construed as a potential conflict of interest.
